# Resection of large, broad-based pedunculated and semi-pedunculated colorectal polyps using a scissor-type endoscopic submucosal dissection knife

**DOI:** 10.1055/a-2788-3249

**Published:** 2026-01-29

**Authors:** Robert Eckersley, Noriko Suzuki, Brian Saunders, Adam Humphries

**Affiliations:** 1 105692 Wolfson Unit for Endoscopy, St Mark's Hospital and Academic Institute, London, United Kingdom of Great Britain and Northern Ireland

**Keywords:** Endoscopy Lower GI Tract, Endoscopic resection (polypectomy, ESD, EMRc, ...), Polyps / adenomas / ..., Colorectal cancer

## Abstract

**Background and study aims:**

Endoscopic resection of large, broad-based pedunculated and semi-pedunculated polyps can be technically difficult. Conventional snare resection risks immediate bleeding and piecemeal excision. We evaluated the safety and efficacy of a scissor-type endoscopic submucosal dissection (ST-ESD) knife for these lesions.

**Patients and methods:**

A single-center retrospective study was conducted of all patients who underwent ST-ESD resection of pedunculated or semi-pedunculated polyps with head size ≧ 20 mm and stalk width ≧ 10 mm. Primary outcomes were en bloc resection, intraprocedural and delayed bleeding, and perforation. Secondary outcomes were R0 resection and recurrence at first follow-up.

**Results:**

Fifty-eight resections were identified (75.6% male; median age 60 years). Median head size was 30 mm (range 20–70) with median stalk width 15 mm (range 10–50). Of them, 74.1% were in the sigmoid colon. Successful resection was achieved in 52 of 58 (89.7%); 44 (75.9%) by ST-ESD alone and eight (13.8%) snare-assisted. Six (10.3%) were abandoned; five due to visible muscle retraction and one dense stalk fibrosis. All successful resections were en bloc with R0 in 52 of 52 (100%). Minor intraprocedural bleeding occurred in nine of 58 (15.5%) and was controlled endoscopically. Forty-seven of 52 defects (90.4%) were closed prophylactically with endoscopic clips. There were no incidences of delayed bleeding or perforation. Histopathology was benign in 51 of 52 (98.1%). One adenocarcinoma was treated surgically for high-risk features. Endoscopic follow-up was available in 41 of 52 (78.8%) with no recurrence seen. Zero of six abandoned cases referred for surgery contained evidence of malignancy in the surgical specimen.

**Conclusions:**

ST-ESD is safe and effective for resection of large, broad-based pedunculated and semi-pedunculated colorectal polyps.

## Introduction

Endoscopic resection of large, broad-based pedunculated and semi-pedunculated polyps presents several challenges. As size increases, so does risk of covert malignancy 1 , making en bloc resection optimal for accurate histopathological evaluation and to reduce recurrence. Traditional en bloc snare polypectomy is often technically difficult due to anatomical and technical factors. A bulky polyp head makes snare manipulation over the whole lesion a difficult and partially blind process, with risk of catching adjacent bowel wall in the snare or performing an inadvertent piecemeal excision. Snare excision of a thick stalk impairs assessment of “muscle retraction,” increasing risk of perforation, while a thick stalk often contains large feeding vessels, increasing risk of immediate bleeding if snare transection is rapid without adequate coagulation. In addition, broad-based polyps are frequently located in the sigmoid colon where the bowel diameter may be narrow and angulated, limiting visibility and scope maneuverability.

Stalk diameter > 5 mm and polyp size > 17 mm are predictors of post-polypectomy bleeding 2 . To mitigate bleeding, various prophylactic methods have been explored. A randomized trial found adrenaline injection into the stalk of polyps ≥ 1 cm reduced immediate bleeding (2% vs 16%) 2 , although a similar study found no significant benefit 3 . Mechanical prophylaxis with detachable snares (endoloops) has shown efficacy in reducing post-polypectomy bleeding in several randomized controlled trials (RCTs) 4 5 6 , although technical difficulty and a failure rate of up to 8.3% have been reported 6 . Mechanical prophylaxis with through-the-scope clips has also been studied; an RCT of 137 polyps ≥ 1 cm showed clips significantly reduced immediate bleeding (odds ratio [OR] 0.22, 95% confidence interval [CI] 0.08–0.57), but had no effect on delayed bleeding 7 . A meta-analysis of 15 RCTs (n = 3,462) confirmed that both adrenaline injection and mechanical prophylaxis reduced early bleeding (adrenaline: relative risk [RR] 0.32, 95% CI 0.11–0.67; clips: RR 0.13, 95% CI 0.03–0.37); however, neither affected delayed bleeding 8 . Moreover, for broad-based stalks, clips and endoloops may obstruct resection or divert cautery current, increasing risk of incomplete removal or inadvertent thermal injury.

Endoscopic submucosal dissection (ESD) has emerged as an alternative to snare resection, enabling controlled, stepwise dissection through the stalk with direct visualization and vessel management. A retrospective study comparing ESD with snare resection for pedunculated polyps ≥ 3 cm reported higher en-bloc resection rates (100% vs 90%) and lower intraprocedural bleeding (4.3% vs 15%) with ESD, without significant differences in perforation or procedure time 9 . In this study, ESD was performed using a needle-type knife with pre-coagulation of vessels using hemostatic forceps.

An alternative approach using a scissor-type ESD (ST-ESD) knife (SB knife, Sumito Baker Light) that has coagulation and cutting capabilities has been described in case reports 10 11 . This knife allows tissue to be grasped, with coagulation applied to vessels before cutting. Its design provides traction and minimizes thermal injury to surrounding tissues due to its insulated blades. Previous studies looking at the safety of efficacy of different ESD knives in resection of non-pedunculated polyps have shown conflicting results, but a recent meta-analysis showed a significant reduction in risk of perforation when using a scissor-type versus a non-scissor-type knife 12 13 . In addition to improved safety, studies have also shown that use of an ST-ESD knife may be better for training, with increased odds of trainee completion without any increase in procedure time when compared with non-ST-ESD knives 13 14 .

Our study presents the largest series to date evaluating outcomes of using ST-ESD knives for large, broad-based pedunculated and semi-pedunculated colorectal polyps.

## Patients and methods

### Study design

This was a retrospective study conducted at a single United Kingdom tertiary referral center evaluating all endoscopic resections of pedunculated and sub-pedunculated polyps greater than 20 mm between January 2015 and December 2023. Consecutive polypectomies performed using a ST-ESD knife were reviewed. Inclusion criteria were pedunculated or semi-pedunculated colorectal polyps (Paris class 0-Ip or 0-Isp) with a head size greater than 20 mm in maximal diameter and a stalk width greater than 10 mm.

Demographic data (referral pathway, age, gender, American Society of Anesthesiologists [ASA] score, antiplatelet/anticoagulant use), polyp characteristics (size, morphology, site), resection technique, intra-procedural events, and post-procedural outcomes were collected from electronic medical records and endoscopy reports.

Primary outcomes assessed were rates of en bloc resection, intra-procedural and delayed bleeding, and perforation. Secondary outcomes included R0 resection rate and recurrence at first follow-up. The study was conducted in accordance with the principles of the Declaration of Helsinki.

### Statistical analysis

Continuous variables were summarized using descriptive statistics, including means, medians, and standard deviations. Categorical variables were reported as frequencies and proportions.

### Endoscopic procedures

All polyps were discussed in a Complex Polyp Multidisciplinary Team (MDT) before resection, with consensus decision to proceed with resection by ST-ESD. This decision was informed by a combination of patient- and polyp-related factors including size, site, morphology, access, suspicion of submucosal invasion, and anticipated adverse events.

All procedures were performed as day-case procedures under either endoscopist-delivered conscious sedation, or anesthetist-delivered propofol deep sedation.

Procedures were performed by three expert therapeutic endoscopists with established competence in endoscopic mucosal resection, needle-type ESD, and scissor-type ESD.

Single-channel adult or pediatric colonoscopes were used. Distal attachments were routinely used, either a short straight cap or conical hood, according to operator preference. Polyp head and stalk size were measured endoscopically, with the open jaws of the SB Knife Jr for refence (diameter 4.5 mm). Submucosal injection was performed in all procedures with a mixed solution of 95 mL 0.9% sodium chloride, 5 mL adrenaline (1:10,000) and 0.5 mL methylene blue. Traction was not used in any case. All resections were performed using an ERBE VIO 2 or 3 electrosurgical generator (ERBE Elektomedezin GmbH, Tübingen, Germany in Endo Cut Q mode with effect 1, duration 3, and coagulation 1. Endoscopic hemostasis was achieved with the scissor-type knife in coagulation mode (VIO 2: forcedCOAG 45W or VIO 3: forcedCOAG 3.5). When hemostasis could not be achieved with the knife, hemostatic forceps were used.

## Results

Fifty-eight resections were identified. Patient demographics and polyp characteristics are summarized in Table 1 . Forty-four of 58 patients (75.6%) were male. Median age was 60 years (range 8–83). Mean ASA score was 1.4 (SD ± 0.7) with 9.4% of patients on antiplatelet or anticoagulant therapy. Fifteen of 58 (26%) were tertiary referrals from other hospitals. Thirty-nine (67.2%) were pedunculated and 19 (32.8%) were semi-pedunculated. Median polyp head size was 30 mm (range 20–70) with median stalk width 15 mm (range 10–50). Forty-three of 58 polyps (74.1%) were in the sigmoid colon.

**Table:** **Table 1** Patient and polyp characteristics.

Patient demographics	Resections (n = 58)
Sex (%)	
Male	44 (75.6)
Female	14 (24.1)
Age (years)	
Median (range)	60 (8–83)
ASA	
Median (range)	1 (1–3)
Anticoagulant/antiplatelet (%)	
Yes	5 (9.4)
No	53 (91.4)
Referral (%)	
Local	43 (74.1)
Tertiary	15 (25.9)
**Polyp characteristics**	
Site (%)	
Cecum	1 (1.7)
Ascending	2 (3.4)
Hepatic	1 (1.7)
Transverse	3 (5.2)
Splenic	0 (0)
Descending	5 (8.6)
Sigmoid	43 (74.1)
Rectum	3 (5.2)
Polyp head size (mm)	
Median (range)	30 (20–70)
Polyp stalk width (mm)	
Median (range)	15 (10–50)
ASA, American Society of Anesthesiologists.	

Procedure outcomes are summarized in Table 2 . Fifty-two of 58 polyps (89.7%) were successfully resected. Six of 58 (10.3%) were abandoned: five due to muscle retraction and one due to dense stalk fibrosis. Forty-four (75.9%) were resected by ESD alone, with eight (13.8%) needing a snare to complete the resection. The 14 incomplete ESD resections are summarized in Table 3 . Two successful ESD resections were completed despite visible muscle retraction, with prophylactic clipping of the muscle layer before continuing with resection. All successful resections were completed en bloc. Minor intra-procedural bleeding occurred in nine of 58 cases (15.5%) and was managed endoscopically with either the ST-ESD knife or hemostatic forceps (Coagrasper, Olympus). Resection defects were closed with clips for 47 of 52 (90.4%) following successful resections. All patients were contacted by telephone in the 2 weeks following the procedure, with no incidences of delayed bleeding or perforation. Histopathology was benign in 51 of 52 successful resections (98.1%), with one of 52 (1.9%) showing adenocarcinoma. R0 resection was achieved in all cases. Six of six abandoned cases (100%) also ultimately showed benign histopathology following surgical resection.

**Table:** **Table 2** Procedure outcomes.

Endoscopic resection	Resections (n = 58)
Technique (%)	
ESD	44 (75.9)
Snare-assisted	8 (13.8)
Abandoned	6 (10.3)
Closure (%)	
Clips	47 (90.4)
Endoloop	1 (1.8)
Nil	4 (7.7)
Histopathological grade (%)	
LGD	28 (53.8)
HGD	18 (34.6)
Adenocarcinoma	1 (1.9)
Other	5 (9.6)
**Complications**	
Intraprocedural bleeding (%)	
Yes	9 (15.5)
No	49 (84.5)
Perforation (%)	
Yes	0 (0)
No	58 (100)
Delayed bleeding (%)	
Yes	0 (0)
No	58 (100)
ESD, endoscopic submucosal dissection; HGD, high-grade dysplasia; LGD, low-grade dysplasia.	

**Table:** **Table 3** Incomplete ESD resections.

Case	Polyp head size (mm)	Polyp stalk width (mm)	Site	Morphology	Endoscopic outcome	Reason	Definitive outcome	Histopathology
A	50	50	Sigmoid	1sp	Abandoned	MRS	Surgery	Peutz-Jegher
B	37	25	Ascending	1p	Snare-assisted	Patient tolerance	–	LGD
C	20	15	Sigmoid	1p	Snare-assisted	Difficult access	–	LGD
D	30	20	Sigmoid	1p	Snare-assisted	Patient tolerance	–	LGD
E	40	25	Sigmoid	1sp	Snare-assisted	Patient tolerance	–	HGD
F	25	15	Sigmoid	1sp	Snare-assisted	Patient tolerance	–	LGD
G	50	25	Sigmoid	1sp	Snare-assisted	Difficult access	–	HGD
H	60	25	Sigmoid	1p	Abandoned	Dense fibrosis	Surgery	HGD
I	35	25	Sigmoid	1sp	Abandoned	MRS	Surgery	LGD
J	45	25	Sigmoid	1p	Snare-assisted	Patient tolerance	–	Adenocarcinoma
K	40	25	Sigmoid	1sp	Abandoned	MRS	Surgery	LGD
L	40	15	Sigmoid	1p	Abandoned	MRS	Surgery	LGD
M	30	15	Transverse	1p	Abandoned	MRS	Surgery	Peutz-Jegher
N	60	30	Sigmoid	1p	Snare-assisted	Difficult access	–	LGD
ESD, endoscopic submucosal dissection; HGD, high-grade dysplasia; LGD, low-grade dysplasia; MRS, muscle retraction sign.								

Endoscopic follow-up data were available for 41 of 52 successful resections (78.8%), with no recurrence seen in any of these cases (median follow-up 36 months). Seven cases were referred for surgery: six abandoned resections that were ultimately benign and one adenocarcinoma with high-risk histopathological features for lymph node metastasis (lymphovascular invasion).

## Discussion

Endoscopic resection of large, broad-based, pedunculated and semi-pedunculated polyps presents significant challenges. Traditional snare polypectomy in these cases carries a heightened risk of piecemeal resection of potentially malignant lesions, as well as increased risks of complications such as bleeding and perforation. To date, there has been limited evidence evaluating the role of ESD in managing these lesions. This retrospective study demonstrates that ESD using a scissor-type knife is both safe and effective for resecting large pedunculated and semi-pedunculated colorectal polyps.

While there is a significant volume of literature looking at endoscopic resection of both large pedunculated and non-pedunculated polyps, there is comparatively little describing techniques for removing large semi-pedunculated lesions. Whether they should be managed with an approach similar to pedunculated or non-pedunculated polyps remains up for debate. The original Paris classification recommends managing as sessile polyps; however, more recent studies have instead grouped thick-stalked pedunculated and semi-pedunculated and pedunculated polyps due to the higher risk of complications, notably post-polypectomy bleeding 9 15 16 . In addition to bleeding, previous studies looking at ESD for large protruding lesions have described the high rates of muscle retraction sign (MRS) (between 9% and 42%) and its associated higher risks of non-curative resection and perforation 17 18 . However, neither of these studies made any distinction between sessile and semi-pedunculated polyps. With no strict definition, in practice the distinction between pedunculated, semi-pedunculated, and sessile polyps is instead a continuum. The experienced therapeutic endoscopist identifies the most likely or serious complications when assessing a polyp and formulating a resection strategy, with difficult access and post-polypectomy bleeding likely to be more common with longer, floppier stalks, whereas muscle retraction and perforation is likely to be more significant with thicker, shorter stalks. In our data, while resection of semi-pedunculated polyps appears to be more challenging, with higher rates of resection abandonment, and intraprocedural bleeding, en bloc resection was achieved in 100% of successful cases, with no recorded incidents of delayed bleeding or perforation. MRS was visible in nine of 58 cases (15.6%). If resected with conventional snare techniques, these would likely have resulted in perforation. Although a small sample size, there was no significant difference between rates of MRS in pedunculated and semi-pedunculated polyps (12.8% vs 21.1%; *P* = 0.45).

This study has several limitations. As a single-center retrospective analysis, there was an inherent risk of selection bias because only procedures in which an experienced endoscopist had already deemed the ST-ESD knife appropriate were included. There was no direct comparison with either conventional snare polypectomy or ESD performed using a needle-type knife. However, the primary objective was to assess safety and efficacy of the ST-ESD knife technique, which this study supports. The study did not address procedure duration because the data collection period predated routine capture of therapeutic procedure times. From 2021 onward, median procedure time was 16 minutes. Although no studies have looked specifically at semi-pedunculated polyps, this procedure time compares favorably with those described in similar studies looking at use of needle-type knives for large pedunculated polyps. In a study of 25 large pedunculated polyps with median head size 30 mm and stalk width 7 mm, Jakobs et al. achieved en bloc resection in 100% of cases with median procedure time of 48 minutes 19 . Similarly, in a study of 29 large pedunculated polyps with mean head size 39.7 mm and stalk width 19.3 mm, Chiba et al. achieved en bloc resection in 28 of 29 cases with mean procedure time 62.6 minutes 20 . Our results suggest that ST-ESD may be significantly faster than needle-type ESD in resecting these polyps. One study comparing needle-type ESD with conventional snare resection for giant pedunculated polyps with head size ≥ 30 mm found no significant difference in procedure times, with mean times of 44.9 and 41.7 minutes for ESD and snare resection, respectively 9 . Generalizability of these results is unclear given the noticeably long procedure time for predominantly en bloc snare resection. Nevertheless, our median procedure time is significantly shorter than both of their procedures. Although it is generally assumed that snare resection is faster than ESD, we hypothesize that the time difference may not be clinically significant for these complex lesions, especially considering the time required to manage any significant intra-procedural bleeding, which is more common with snare resection. Undoubtedly, accurate recording of procedure times would be a key outcome of any future prospective studies. The follow-up rate was relatively low at 78.8%; however, this must be interpreted in context, because a substantial proportion of cases have not yet reached the surveillance interval recommended by European guidelines - for patients with an R0 resection and no additional polyps, current guidance advises a follow-up colonoscopy at 3 years 21 . In addition, as a tertiary center with a broad referral base, many patients were discharged to their local hospitals after successful resection of benign lesions for continued surveillance locally, contributing to the lesser amount of follow-up data available.

The place of this technique within the expanding toolkit of therapeutic endoscopy remains to be clearly defined. A prospective, comparative study evaluating outcomes between conventional snare and ST-ESD knife resection for large, broad-based pedunculated and semi-pedunculated polyps would be instrumental in guiding optimal management. Moreover, future prospective studies should record procedure duration, intra-procedural events, and hemostatic interventions to provide a more comprehensive comparison.

As for all forms of polypectomy, accurate lesion assessment and selection of the most appropriate technique is a key factor for determining successful resection. Assessing large pedunculated and semi-pedunculated polyps can be particularly challenging due to limited visibility and difficult access, often compounded by trauma to the polyp head that obscures surface pattern analysis, and artificial intelligence (AI) and computer-aided diagnosis (CADx) hold promise for augmenting accurate lesion assessment. Algorithms trained on large datasets may enable accurate prediction of submucosal invasive cancer risk; furthermore, CADx systems could develop the ability to assess likelihood of complications such as bleeding or perforation. Together, these advances may support more accurate lesion assessment and inform better selection of resection techniques.

## Conclusions

In conclusion, this is the largest study to date evaluating ST-ESD knife resection for large, broad-based, pedunculated and semi-pedunculated polyps. Our findings support the safety and efficacy of this technique, although further prospective, comparative studies are needed to define its role and place in the therapeutic endoscopist’s toolkit.
